# A New Deep Learning Algorithm for SAR Scene Classification Based on Spatial Statistical Modeling and Features Re-Calibration

**DOI:** 10.3390/s19112479

**Published:** 2019-05-30

**Authors:** Lifu Chen, Xianliang Cui, Zhenhong Li, Zhihui Yuan, Jin Xing, Xuemin Xing, Zhiwei Jia

**Affiliations:** 1 School of Electrical and Information Engineering, Changsha University of Science & Technology, Changsha 410114, China; Lifu.Chen@newcastle.ac.uk (L.C.); cxl@stu.csust.edu.cn (X.C.); jiayege@csust.edu.cn (Z.J.); 2 School of Engineering, Newcastle University, Newcastle upon Tyne NE1 7RU, UK; Zhenhong.li@newcastle.ac.uk (Z.L.); Jin.Xing@newcastle.ac.uk (J.X.); 3 Laboratory of Radar Remote Sensing Applications, Changsha University of Science & Technology, Changsha 410014, China; xuemin.xing@csust.edu.cn; 4 College of Geological Engineering and Geomatics, Chang’an University, Xi’an 710054, China; 5 School of Traffic & Transportation Engineering, Changsha University of Science & Technology, Changsha 410114, China

**Keywords:** SAR, scene classification, deep learning, convolutional neural network (CNN), attention mechanism, multi-scale spatial feature, Gabor Transform, gray-level gradient co-occurrence matrix (GLGCM), gaussian derivative filter

## Abstract

Synthetic Aperture Radar (SAR) scene classification is challenging but widely applied, in which deep learning can play a pivotal role because of its hierarchical feature learning ability. In the paper, we propose a new scene classification framework, named Feature Recalibration Network with Multi-scale Spatial Features (FRN-MSF), to achieve high accuracy in SAR-based scene classification. First, a Multi-Scale Omnidirectional Gaussian Derivative Filter (MSOGDF) is constructed. Then, Multi-scale Spatial Features (MSF) of SAR scenes are generated by weighting MSOGDF, a Gray Level Gradient Co-occurrence Matrix (GLGCM) and Gabor transformation. These features were processed by the Feature Recalibration Network (FRN) to learn high-level features. In the network, the Depthwise Separable Convolution (DSC), Squeeze-and-Excitation (SE) Block and Convolution Neural Network (CNN) are integrated. Finally, these learned features will be classified by the Softmax function. Eleven types of SAR scenes obtained from four systems combining different bands and resolutions were trained and tested, and a mean accuracy of 98.18% was obtained. To validate the generality of FRN-MSF, five types of SAR scenes sampled from two additional large-scale Gaofen-3 and TerraSAR-X images were evaluated for classification. The mean accuracy of the five types reached 94.56%; while the mean accuracy for the same five types of the former tested 11 types of scene was 96%. The high accuracy indicates that the FRN-MSF is promising for SAR scene classification without losing generality.

## 1. Introduction

With the fast development of remote sensing technology, the variety of the acquired imagery datasets has been increasing, such as Hyperspectral images, Light Detection And Ranging (LiDAR) dense point clouds, and Synthetic Aperture Radar (SAR) images with different bands. The volume and complexity of skyscraping data requires automatic interpretation of remote sensing images, which has become an urgent task to achieve the goal of a digital earth [1]. Among numerous image interpretation techniques, scene classification using remote sensing images attracts considerable research interest. For decades, scene classification has been widely applied in many fields such as natural disaster monitoring [2,3,4,5], land use and land cover classification [6,7], target detection [8,9,10], geographical space targets monitoring [11], geographical images search [12], vegetation mapping [13], environment monitoring and city planning [14]. SAR is an active earth observation system that offers all-day and almost all-weather advantages over other sensors. These advantages make SAR scene classification more appealing. However, there are three unique challenges in SAR scene classification. First, speckle noise in SAR imagery is more complicated than the noise in other remote sensing data, because speckle is a granular noise that inherently exists and reduces the quality of SAR images [15]. Second, there is not yet a deep learning network which is specifically designed for SAR image classification. The networks commonly used are all directly relevant to optical images, which cannot be directly applied to SAR images. Third, the generalization of SAR scene classification algorithms is more complicated, as the variations in image parameters (such as frequency, spatial resolution and temporal resolution) are much greater than those of optical images. 

The contributions of this paper are as follows:

(1) A framework for SAR scene classification is proposed, which contains two parts: multi-scale spatial features (MSF) extraction and Features Recalibration Network (FRN). The first part aims to extract multi-scale low-level features, while the second part intends to extract high-level features and then confirm the types of targets.
(2) An example of the MSF is presented, in which a Multi-Scale Omnidirectional Gaussian Derivative Filter (MSOGDF), a Gray Level Gradient Co-occurrence Matrix (GLGCM) and Gabor transformation are used to extract rich detailed information from SAR images.
(3) An example of the FRN is demonstrated, in which Depthwise Separable Convolution (DSC), attention mechanism and CNN are integrated to better extract the high-level features of different types of targets.
(4) A prototype of the above-mentioned framework is implemented and its performance is assessed using SAR data with different frequency bands and different resolutions.

The remainder of the paper is organized as follows. Section 2 introduces the framework proposed in detail, mainly two parts, MSF and FRN. Section 3 gives the experiments and the corresponding results, which validate the accuracy and the generalization of the proposed framework. Section 4 gives a discussion on the weighting selection for different features followed by some conclusions.

## 2. Background

### 2.1. State of the Art

Many scene classification approaches have been proposed, which can be categorized into three types according to the features extraction [16]: (1) methods based on handcrafted-feature, which usually use expertise and engineering skills to extract useful information to distinguish between different types of targets [17], such as texture, shape and spectral features; (2) methods based on unsupervised-feature-learning, which extract more discriminative features than manually designed features by learning from unlabeled input data automatically [18,19]; (3) methods based on deep-feature-learning, which extract high-level features of targets by learning from labeled input data [20,21], as proposed by Hinton and Salakhutdinov in 2006 [22].

Deep learning extracts image features through a multi-layer neural network. As the number of the network layers increases, it can extract higher-level features and thus better interpret SAR images. It stems from an artificial neural network, which integrates low-level features to form abstract high-level features to determine the types of different targets. The low-level features mainly embody the detailed information of targets, such as the texture and edges; while the high-level features mainly reflect the features of types, which is better for classifying targets. Since then, deep learning has been studied and used by scholars in many fields, especially in optical image processing (such as target detection, object recognition and image segmentation). Hinton et al. [23] presented a fast, greedy algorithm which used “complementary priors” to learn deep, Directed Belief Networks (DBN) one layer at a time. It achieves better digit classification result than the best discriminative learning algorithm, which brings some hope to solve the optimization problem of deep structures. In the ImageNet contest of 2012, Krizhevsky et al. used a Convolutional Neural Network (CNN) to achieve a 10% accuracy improvement [24]. It was the first time that the performance of deep learning exceeded those of traditional learning modes, in which the features are manually designed and shallow networks are used in the training mode. Since then, the upsurge for deep learning has begun. In 2016, AlphaGo, an artificial intelligence robot developed by DeepMind, defeated the top player of mankind in the game Go [25]. Many deep learning algorithms and Monte Carlo searching were adopted in it. The learning mechanisms of deep learning are identical to machine learning, including supervised learning and unsupervised learning.

At present, there does not exist a sophisticated deep learning framework for SAR image analysis. Due to the noticeable progress of deep learning in optical image processing [26], SAR scene classification largely adopts algorithms from optical image classification. Successful examples include the Bag-of-Visual-Words (BoVM) [27], the unsupervised probabilistic Latent Semantic Analysis (pLSA) model [28] and the k-Nearest Neighbor (k-NN) classifier [29]. However, most research favors developing SAR specific scene classification methods. For example, Cloude and Pottier [30] combined polarimetric SAR modeling and entropy-based classifier for SAR-based scene classification. But these methods largely depend on middle-level or low-level SAR features. Sheng et al. [31] proposed Local Ternary Pattern Histogram Fourier (LTP-HF) transformation to extract SAR features, while Cheng et al. [32] and Hu et al. [33] constructed BoVM to encode local features. However, these SAR features normally require manual selection, which fail to accommodate the scene classification task in terms of high-level semantic feature representation or big data challenge. 

Scientists have begun to investigate classification methods that fill in the gap between low-level features and high-level features. Selim et al. [34] constructed a Bayesian classifier based on visual grammar learning to perform scene classification. This method could achieve satisfactory accuracy, but could not fully learn the rich high-level information of SAR images, but deep learning can solve this problem effectively. Deep learning approaches can extract high-level semantic features from SAR images hierarchically, which can encode semantic information of the scene more effectively. Zhong et al. [35] combined traditional local features expression with CNN to tackle scene classification, which automatically builds high-level image features using local ones. Hu et al. [36] proposed sparse filter to learn features of salient regions, which were employed to parameterize the convolutional layers within CNNs, for SAR scene classification. 

However, none of the previous work has explored the fusion of high-level SAR features with deep learning in SAR scene classification. Therefore, we propose a more sophisticated deep learning framework to achieve higher accuracy by integrating high-level SAR features with low-level features automatically.

### 2.2. Deep Learning

Deep learning is a new field derived from machine learning, which aims to analyze various datasets by simulating human brain processing of targets, such as images, voices and texts. CNN builds hierarchical networks to process data with given grid-like structures [37]. It relies on convolutional kernels to learn structural features through multiple layers of neurons hierarchically. The learnt features are usually passed to classifiers such as Softmax [38], to form the complete workflow of supervised learning. The three fundamental layers of CNN are shown as follows:

(1) Convolutional layer: it is the core of a CNN, in which the convolution operation is employed to replace matrix multiplication. Supposing that the input image is a m×n matrix X and the number of convolutional kernels is K with a dimension of k×k, the dimension of the output matrix Y is ((m−k)/s+1)×(n−k)/s+1) after convolution as follows:

(1) $$y_{i}=b_{i}+\underset{i}{\sum }\omega_{ij}⊗x_{i}$$

where $x_{i}$ is the input of the front layer, $\omega_{ij}$ is the weight, $b_{i}$ is the bias and s is the stride of the convolutional kernel.

(2) Activation layer: this layer implements non-linear transformation(s) to the input:

(2) $$f_{s}=\tau \left(b_{i}+\underset{i}{\sum }\omega_{ij}⊗x_{i}\right)$$

where *τ* is the activation function, with the commonly used ones being Sigmod, Tanh, and Rectified Linear Units (ReLU) [37]. ReLU can improve the sparse expression ability of the network and prevents over-fitting. It solves the problem of gradient dissipation caused by the increasing number of network layers and accelerates the convergence of Stochastic Gradient Descent (SGD) [37]. Therefore, this paper employed ReLU as the activation function.

Pooling layer: when the input image is big, feature maps produced by the convolution layers are huge and contain considerable superfluous features. To solve this redundancy problem, a pooling layer is used to down-sample the feature maps. The most commonly used pooling strategies are Max-Value, Mean-Value and Root-Mean-Square [37].

## 3. Methodology

### 3.1. The Framework Architecture

**Figure 1:**
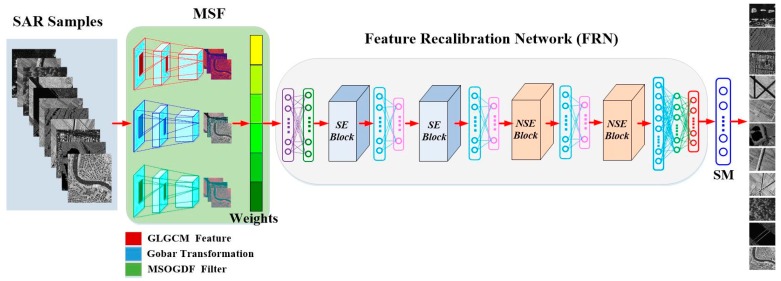
The proposed framework architecture.

The proposed framework architecture is illustrated in Figure 1. First, training samples and testing samples are generated respectively. Second, training samples are processed by the Multi-scale Spatial Feature (MSF) module. In MSF, the Gray Level Gradient Co-occurrence Matrix (GLGCM) features, Gabor transformation and Multi-scale Omnidirectional Gaussian Derivative Filter (MSOGDF) are employed to extract the corresponding low-level features. Then, different weights for the three features are assigned according to the analysis of classification performance. Third, the fused image is analyzed using the proposed Feature Recalibration Network (FRN) to extract high-level features for scenes. In FRN, it includes two Squeeze-and-Excitation (SE) Blocks [36], two New Squeeze-and-Excitation (NSE) Blocks, some convolution layers, pooling layers and full connection layers. In the proposed NSE Block, Depthwise Separable Convolution (DSC), SE Block and CNN are integrated to produce an enhanced module for classification. Finally, the Softmax function classifies these enhanced feature maps as pre-defined scene types.

### 3.2. Multi-Scale Spatial Feature (MSF)

MSF aims to acquire multi-scale spatial statistical features of SAR images. This is inspired by [39], in which integration of various features extracted from multi-scale SAR images could improve the accuracy of classification. In this paper, we developeda method of GLGCM, Gabor transform and MSOGDF to extract different features of SAR images, which are then fused to learn high-level features.

#### 3.2.1. GLGCM Extraction

GLGCM reflects the relationship of two basic elements in an image, gray level and gray gradient. The gray level stands for the pixel values of a given SAR image, while the gray gradient is the directional change in grey levels, which are commonly employed for image edge detection and texture analysis [40]. GLGCM can describe the texture of the given image, in which the directional texture can be depicted by the direction of the gray gradient [41].

Supposing SAR image is f(i,j), i,j=0, 1,2, …, N−1. *N* is the dimension size of the image.

(1) Normalization processing for the image: $F\left(i,\;j\right)=\left[f\left(i,j\right)L_{f}/f_{max}\right]+1$. $L_{f}$ is the total number of gray levels, and $f_{max}$ is the maximum gray value of a given SAR image.
(2) Computing the gray gradient image: g(i, j), i,j=0, 1,2, …, N−1.
  To better extract texture information, four Sobel operators in four directions with a 3 × 3 window are adopted considering the amount of calculation. They are 0°, 45°, 90° and 135°, which are denoted by $S_{0}$, $S_{45}$, $S_{90}$, and $S_{135}$ respectively.
  (3) $$S_{0}=\left[\begin{array}{ccc} -1 & -2 & -3\\ 0 & 0 & 0\\ 1 & 2 & 1 \end{array}\right],\;S_{45}=\left[\begin{array}{ccc} -2 & -1 & 0\\ -1 & 0 & 1\\ 0 & 1 & 2 \end{array}\right],\;S_{90}=\left[\begin{array}{ccc} -1 & 0 & 1\\ -2 & 0 & 2\\ -1 & 0 & 1 \end{array}\right],\;S_{135}=\left[\begin{array}{ccc} 0 & 1 & 2\\ -1 & 0 & 1\\ -2 & -1 & 0 \end{array}\right]$$
  Then, the gradient value g(k, l) of the pixel (k, l) is computed by Equation (4):
  (4) $$g\left(k,\;l\right)=\sqrt{g_{0}^{2}+g_{45}^{2}+g_{90}^{2}+g_{135}^{2}}$$
  (5) $$g_{m}=f{\left(k,\;l\right)}_{3\times 3}\;\times S_{m},\;m=0,\;45,\;90,\;135$$
  where the symbol ∗ means dot product operation between two matrices, and $f{\left(k,\;l\right)}_{3\;\times \;3}$ denotes 3 × 3 matrix values around the central pixel (k, l).
(3) Gray gradient image normalization: $G\left(i,\;j\right)=\left[g\left(i,j\right)L_{g}/g_{max}\right]+1$. $L_{g}$ is the number of gray levels for the gray gradient image, and $g_{max}$ is the maximum value of the gradient matrix.
(4) GLGCM computation: $H\left(i,\;j\right):\;i=0,\;1,2,\;\ldots ,\;L_{f}-1,\;j=0,\;1,2,\;\ldots ,\;L_{g}-1$. It counts the number of the point pairs in the image which satisfies f(m1, n1)=i, g(m2,n2)=j, simultaneously.

Fifteen commonly used quadratic statistical characteristics can be computed based on the normalized GLGCM, including small gradient dominance, large gradient dominance, inhomogeneity of gray distribution, inhomogeneity of gradient distribution, energy, gray mean, gradient mean, gray mean square error, gradient mean square error, correlation, gray entropy, gradient entropy, mixing entropy, inertia and homogeneity [40]. Then the characteristics of correlation and inertia are selected in the paper after further analysis.

#### 3.2.2. Gabor Transformation

Gabor transform is much like the visual stimulating response of simple cells in human visual system. It has sound performance in extracting local spatial information and frequency features of targets. Compared with traditional Fourier transformation, Gabor transformation has better performance in time-frequency domain analysis. We usually adjust the directions, baseband bandwidth and central frequency of the Gabor filter to better tackle the resolution of signal in both spatial-temporal domain and frequency domain [42].

This paper adopts a two-dimensional Gabor filter: $G\left(x_{0},y_{0},\theta ,\omega \right)$ [43], to perform convolution with SAR images: I(x,y).

(6) $$IG=I\left(x,y\right)⊗G\left(x_{0},y_{0},\theta ,\omega \right)$$

(7) $$G\left(x_{0},y_{0},\theta ,\omega \right)=\frac{1}{2\pi \sigma^{2}}\exp\left(-\frac{x_{0}^{2}+y_{0}^{2}}{2\sigma^{2}}\right)\cdot \left[\exp\left(j\omega x_{0}\right)-\exp\left(-\frac{\omega^{2}\sigma^{2}}{2}\right)\right]$$

where ⊗ denotes convolution operation, where $x_{0}=xcos\theta +ysin\theta ,\;y_{0}=-xsin\theta +ycos\theta ,$ and θ is the directional angle. ω is the central frequency of the filter and σ is the mean square error of Gauss function. $\exp\left(j\omega x_{0}\right)$ is the alternating component, and $\exp\left(-\frac{\omega^{2}\sigma^{2}}{2}\right)$ denotes the direct component [39].

The features extracted by Gabor transformation are mainly local texture information. These features depend on the Gabor kernel, which acts as a sliding window in the frequency domain to extract local information. In this paper, Gabor transformation in the direction of 45° and 135° were selected, because they can better represent the features of SAR image in the experiments than other directions.

#### 3.2.3. Multi-Scale Omnidirectional Gaussian Derivative Filter (MSOGDF)

Generally, the global visual spatial structure of images follows Weibull distribution statistically. Therefore, the Weibull model of global spatial structure of images can be constructed to represent the visual characteristics of images. The local spatial structure of each pixel can be represented by Taylor expansion of the image I(x,y) at the given point. Thus, the observation value $\hat{I}\left(x,y\right)$ of the SAR image is given by the Taylor approximation in Equation (8):

(8) $$\hat{I}\left(x,y\right)=\hat{I}{\left[\begin{array}{c} x\\ y \end{array}\right]}^{T}\left[\begin{array}{c} I_{x}\\ I_{y} \end{array}\right]+\frac{1}{2}{\left[\begin{array}{c} x\\ y \end{array}\right]}^{T}\left[\begin{array}{c} I_{xx\;}\;I_{xy}\\ I_{yx}\;I_{yy} \end{array}\right]\left[\begin{array}{c} x\\ y \end{array}\right]+\cdots$$

It indicates the observation value is obtained by accumulating the spatial structure information of the image at a given spatial scale. This illustrates that the most important visual characteristics of an image are determined by the spatial structure of the image. The differential term $I_{x^{m}y^{n}}$ (m,n=0,1,2,⋯) represents the spatial structure characteristics of the image, which can be generated by a Gaussian derivative filter $G_{k,\sigma }\left(x,y,\sigma \right)$ [44]:

(9) $$I_{x^{m}y^{n}}\left(x,y\right)=I\left(x,y\right)G_{k,\sigma }\left(x,y,\sigma \right)$$

where k=m+n is the order of the filter, and σ is the scale parameter.

We can only obtain filtered SAR images in the direction of x and y via Equation (9). To tackle this problem, we developed an omnidirectional Gaussian derivative filter to extract spatial structure features with arbitrary directions θ. The omnidirectional Gaussian derivative filter $G_{k,\sigma }^{\theta }\left(x,y\right)$ is shown in Equation (10):

(10) $$G_{k,\sigma }^{\theta }\left(x,y\right)=\overset{M}{\underset{i=1}{\sum }}k_{i}\left(\theta \right)G_{i_{k,\sigma }}^{\theta }\left(x,y\right)$$

where M is the number of filter bases $G_{i_{k,\sigma }}^{\theta }\left(x,y\right)$, and $k_{i}\left(\theta \right)$ is the interpolation function. 

In polar coordinates, supposing $\gamma =\sqrt{x^{2}+y^{2}}$ and ψ=arg(x,y), then the Fourier series of $G_{k,\sigma }^{\theta }\left(\gamma ,\psi \right)$ can be expanded in polar angle ψ:

(11) $$F\left\{G_{k,\sigma }^{\theta }\left(\gamma ,\psi \right)\right\}=\overset{N}{\underset{n=0}{\sum }}a_{n}\left(\gamma \right)e^{in\psi }$$

To solve Equation (10) by using Equation (11), if and only if $k_{i}\left(\theta \right)$ are solutions of the following equation [44]:

(12) $$\left(\begin{array}{c} 1\\ e^{i\theta }\\ \vdots \\ e^{in\theta } \end{array}\right)=\left(\begin{array}{cccc} 1 & 1 & \cdots & 1\\ e^{i\theta_{1}} & e^{i\theta_{2}} & \cdots & e^{i\theta_{M}}\\ \vdots & \vdots & \vdots & \vdots \\ e^{in\theta_{1}} & e^{in\theta_{2}} & \cdots & e^{in\theta_{M}} \end{array}\right)\left(\begin{array}{c} k_{1}\left(\theta \right)\\ k_{2}\left(\theta \right)\\ \vdots \\ k_{M}\left(\theta \right) \end{array}\right)$$

If, for any n, $a_{n}\left(\gamma \right)=0$, then the corresponding (nth) row of the left-hand side and of the matrix of the right-hand side of Equation (12) should be removed.

In this paper, a Multi-Scale Omnidirectional Gaussian Derivative Filter (MSOGDF) has been proposed, using 6 directions between 0 and π with an interval of 30°, to extract the features of the targets.

#### 3.2.4. Multi-Scale Spatial Features (MSF) Fusion

Features extracted by GLGCM, Gabor and MSOGDF contain rich low-level information of the targets, which are very helpful for the framework to confirm detailed information of different types of scenes. Then, these features are fused by a weighted vector as the input to the CNN, to generate higher level features via convolution layers and pooling layers. This process also removes redundant features. The weight vector is generated according to the classification performance of these features for different types of scenes.

### 3.3. Depthwise Separable Convolution (DSC)

**Figure 2:**
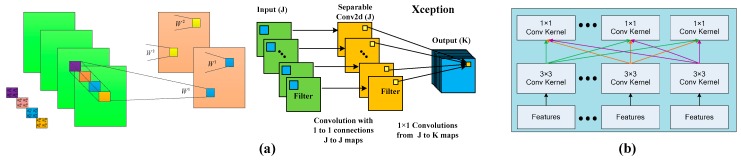
Convolution module (**a**), Standard convolution module (**b**), Depthwise separable convolution module.

CNNs have demonstrated their prominence in the field of image recognition. The most straightforward way to improve network performance is to increase the depth of the network, such as with the Visual Geometry Group-19 (VGG-19) model [45], which can enhance the network’s ability for characterizing data features. However, the more network layers, the greater the number of parameters, which makes the convergence more challenging [46]. In order to solve this problem, a deep separable convolution module [46] is employed to replace additional convolution layers with more parameters. The schematic diagram of the convolution module is shown in Figure 2.

As shown in Figure 2a, a 2 × 2 convolutional kernel was been applied to the input image. Supposing there is a 3 × 3 convolution layer with 16 input channels and 32 output channels, we employ 32 convolutional kernels (each kernel is 3 × 3) to process the input data. Thus, each convolutional kernel brings 3 × 3 × 16 parameters, and the 3 × 3 × 16 convolutional kernel combines 16 input channels to generate a single channel output. Therefore, the number of parameters for 32 convolution kernels is 4068 (i.e., 3 × 3 × 16 × 32).

The DSC module divides the convolutional operation into two steps: (1) Depthwise operation uses 16 convolutional kernels and each kernel is 3 × 3 (one channel), from which we obtain 16 feature maps from 16 input channels; (2) Separable operation utilizes 32 convolution kernels and each kernel is 1 × 1 (16 channels), to fuse 16 feature maps. The whole process includes 656 (3 × 3 × 16 + (1 × 1 × 16) × 32) parameters. 

Compared with standard convolution operations, depth separable convolution reduces the number of the required parameters significantly. More importantly, DSC handles regions and channels separately.

### 3.4. New Squeeze-And-Excitation (NSE) Block

The recognition mechanism of convolutional neural networks imitates the recognition process of the human brain. In the process of recognition, the human cerebral cortex has different levels of excitement for different targets, which is called the attention mechanism. This inspires the attention mechanism of computer vision [47]. This mechanism guides the neural network to learn the important levels of features within the entire recognition task. It has been applied well in text translation [48], text matching [49], speech recognition [50], and especially image recognition [51]. SE Block proposed by He et al. [36] is a deep learning method, which is a prominent implementation of attention mechanism.

**Figure 3:**
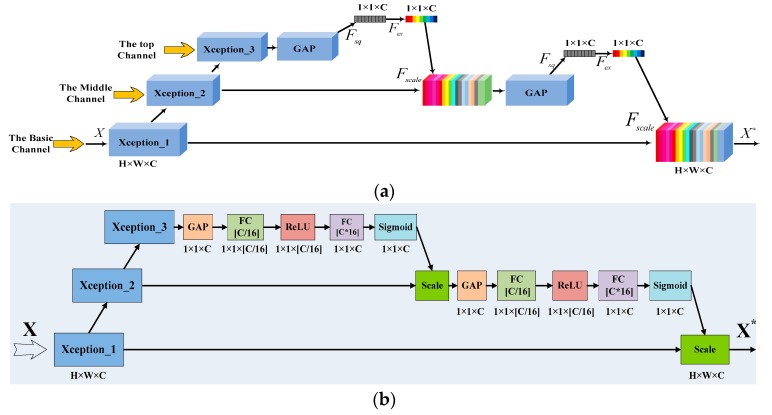
The structure of the New Squeeze-and-Excitation (NSE) Block. (**a**) the workflow of the NSE block, (**b**) the specific layer setting for the workflow of the NSE block.

In this paper, a New Squeeze-and-Excitation (NSE) Block is proposed, integrating a SE Block, and CNN with a DSC, as illustrated in Figure 3. This is an enhancement module to extract the high-level features of different types of targets. We developed our DSC module based on Xception [46]. 

As demonstrated in Figure 3b, a feature map with the size of *H* × *W* and *C* channels of the input X is processed by the DSC layer, Xception_1, initially. Then, high-level features are extracted through Xception_2 and Xception_3. The feature map outputted by Xception_3 with *C* feature maps is further handled by Global Average Pooling (GAP):

(13) $${\bar{x}}_{i}=GAP\left(x_{i}\right)=\left(\overset{W}{\underset{j=1}{\sum }}\overset{H}{\underset{i=1}{\sum }}a_{ij}\right)/\left(H\times W\right)$$

where $x_{i}$ and ${\bar{x}}_{i}$ are the feature maps before and after GAP respectively, and $a_{ij}$ is the value of each pixel in the feature map. 

By this means, the correlation among channels is tackled by two Fully Connected (FC) layers with a bottleneck layer in the middle [36]. A weighted vector with dimension *C* is generated by the second FC layer, and then it is normalized via a Sigmoid function:

(14) $$\left({\tilde{x}}_{1},{\tilde{x}}_{2},\cdots ,{\tilde{x}}_{C}\right)=sig\left[\sigma \left({\tilde{x}}_{1},{\tilde{x}}_{2},\cdots ,{\tilde{x}}_{C}\right)\right]$$

where σ denotes the bottleneck operation, and sig denotes the normalization for the vectors, while $\left({\tilde{x}}_{1},{\tilde{x}}_{2},\cdots ,{\tilde{x}}_{C}\right)$ is the normalized weight vector. 

The SE Block only considers the relationship among feature maps at the given layer, rather than that between two layers. Therefore, we need a high-level feature to capture such relationship. To achieve this, the weighted integration of the top and middle channel is proposed, which is added to the low channel via the scale operation:

(15) $$x_{i}^{*}={\tilde{x}}_{i}\cdot x_{i}$$

Therefore, the more effective features can be extracted fully by weighting of different channels, through the NSE Block. It can suppress the redundant features which affect the classification accuracy and perform the recalibration of the multi-scale features. Then, it can improve the classification accuracy finally.

In the paper, a framework is proposed to carry out scene classification, which is called Feature Recalibration Network with Multi-scale Spatial Features (FRN-MSF). At first, it extracts rich low-level features by the MSF module, which can better describe the detailed information of the targets. Then, the fused features are the inputs to the network FRN, which integrates SE Blocks, the proposed NSE Blocks, some convolution layers, pooling layers and fully connected players. It can better extract high-level features, which can in turn be used to confirm the types of targets.

## 4. Experiments and Results 

### 4.1. Datasets Used in this Study

To assess the proposed framework, SAR images with different bands, resolutions, and acquired from different platforms were utilized. The dataset contains two images from TerraSAR-X, five images from Gaofen-3, one image from airborne millimeter InSAR (MM-InSAR) and one image from airborne X-band InSAR (CAS-InSAR), as shown in Table 1. SAR images used in these systems are all single polarization products. The two TerraSAR-X images were acquired from the Dongtinghu and Foshan areas, respectively. The five Gaofen-3 images covered five airports (i.e., Shanghai airport). The very-high-resolution (VHR) MM-InSAR image was acquired at Xi’an, China. The CAS-InSAR image was provided by the Chinese Academy of Science, and we selected an image taken at Weinan, China. We used nine large-scale SAR images in total. Seven images were selected for training and testing of the framework except for the Foshan image from TerraSAR-X and the one airport image (Carstensen airport) from Gaofen-3. To evaluate the generalization ability, we utilized the trained model to test samples selected from Foshan image and the unused airport image.

**Figure 4:**
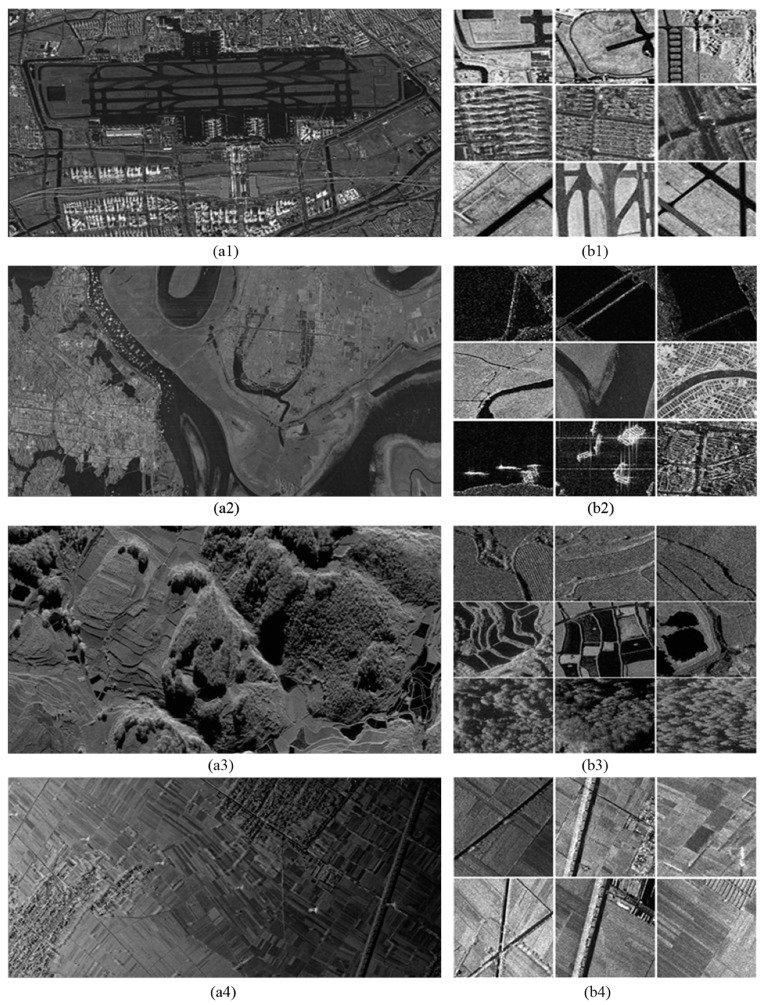
SAR images and samples: (**a1**–**a4**) SAR images used in the paper; (**b1**–**b4**) The scene samples extracted from left SAR images.

Samples of the datasets are shown in Figure 4. Figure 4a1–a4 illustrate parts of the sampled SAR images acquired at Shanghai, Dongtinghu, Xi’an and Weinan SAR images. Figure 4b1–b4 show samples extracted from the corresponding SAR images in Figure 4a1–a4. 

**Figure 5:**
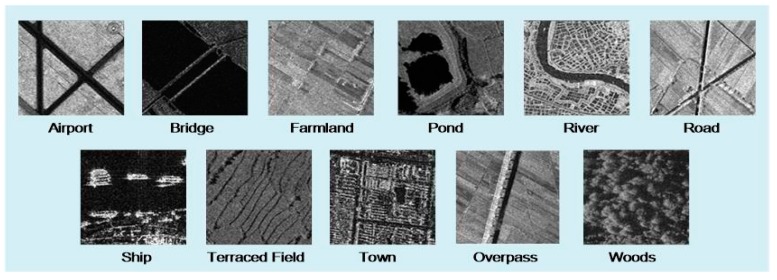
Examples of the 11 types of scenes.

A total of 300 samples for each type were generated with a size of 500 × 500 pixels by the commonly used method for preparing samples in the remote sensing image scene classification [1]. A total of 11 kinds of scenes were generated in total, including airport, bridge, farmland, pond, river, road, ship, terraced field, town, overpass and woods. In the datasets, 80% of the samples for each type were selected randomly as training samples, and the rest are selected as testing samples. Examples for the 11 types are shown in Figure 5. In the airport samples, airplanes were not included, but airport runways, grassland and aprons were. The bridge samples contained only bridges above water. The pond samples were small pools, rather than lakes. The roads we selected did not contain urban roads, which were marked as town. The ship samples were only ships in water. The overpass samples only contained bridges across roads in the city.

### 4.2. Parameters Setting of the Proposed Framework

For the SAR datasets, FRN was proposed to perform classification. It contained four Conv2D convolution layers, four pooling layers, two NSE Blocks, two SE Blocks, three FC layers and one Softmax layer. The multi-scale fusion images were the input of the network. The output results of the network were normalized vectors in accordance with pre-defined scene types. The results were generated by the Softmax layer. The parameters of the network are shown in Table 2.

### 4.3. Results

#### 4.3.1. Multi-Scale Feature Extraction and Fusion

In this paper, three feature extraction methods were used, including GLGCM, Gabor transformation and MSOGDF. For a SAR scene, we could compute 15 digital feature maps by GLGCM. After analyzing the effects of texture features for the 11 types of scene, the correlation and inertia features were selected.

**Figure 6:**
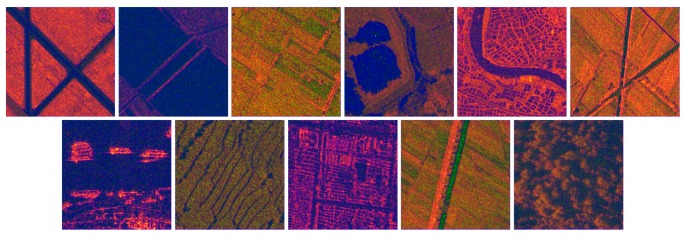
Fusion map of correlation and inertia for the 11 types of scenes.

The correlation and inertia GLGCM features were further combined as a fused feature for SAR scene classification. The correlation describes the grayscale similarity between rows and columns in a matrix, which is a measure of the relationship between gray level and gray gradient in GLGCM. The greater the similarity, the higher the correlation coefficient is. While the inertia reflects the smoothness of the texture. The coarser the texture is, the smaller the inertia value is. Fusing the two features together with SAR image by concatenation [52], we could obtain the enhanced texture feature, as depicted in Figure 6. 

**Figure 7:**
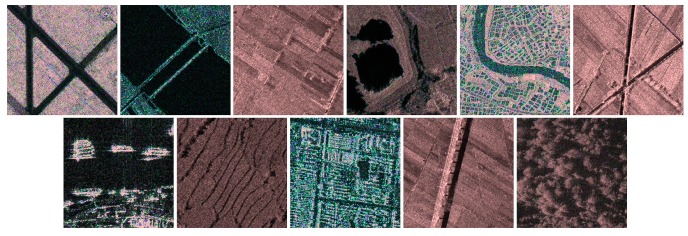
The fusion map of two Gabor features for the 11 types of scenes.

The Gabor function is like the biological function of the human eye, which is frequently used to recognize a texture and achieves good results. Through the analysis for four angles of feature extraction with Gabor transformation in the experiments, we found that the 45° and 135° transformation features were clearer. We could get the fusion map in Figure 7 after fusing the 45° and 135° features together. From which, we could see much better local texture features, especially the edge information. It is much more useful for us to classify the different types of scene.

According to Equation (10), we analyzed the feature effects of the different directions for θ by 30° spacing with the scale of σ is 1 or 2 respectively. Finally, we selected three directions (45°, 90° and 135°) with two scales (σ=1,2), considering the characteristic of the targets in different directions and the demand of the three channels in the concatenation method. Therefore, we could generate the six features of a SAR image.

**Figure 8:**
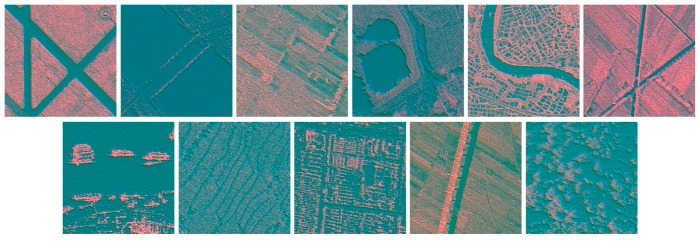
The fusion map of two feature maps of MSOGDF for the 11 types of scenes.

MSOGDF can extract multi-directional gray-scale change information, which is frequently applied to identify edges and corners. It can capture detailed spatial information with different directions in the image. Thus, it is widely employed to classify various objects [44]. Figure 8 delineates the fusion map of two feature maps of MSOGDF in 90° direction with scale σ=1 and σ=2. The fusion map presents more detailed information about targets. 

#### 4.3.2. Performance Assessment

(1) Feature Fusion Method

In this paper, we use three methods to extract different features of the targets: GLGCM, Gabor and MSOGDF. For GLGCM, we fused the correlation and inertia feature maps with the SAR image into a three-channel matrix as the output. As Gabor transformation is concerned, we combined the feature maps of 45° and 135° with the SAR image into a new three-channel feature map. For MSOGDF, we select three directions, namely 45°, 90°and 135°. For each direction, we chose two observation scales, σ=1 and σ=2. The feature maps were grouped by angle, within each group two different scale feature maps were integrated to form a three-channel matrix as the new feature map. 

For MSOGDF, the three groups of fusion features were separately experimented for classification with the proposed algorithm. From Table 3, we find that the 45° group presented the best classification results. Therefore, we selected this group as the input for further analysis.

**Figure 9:**
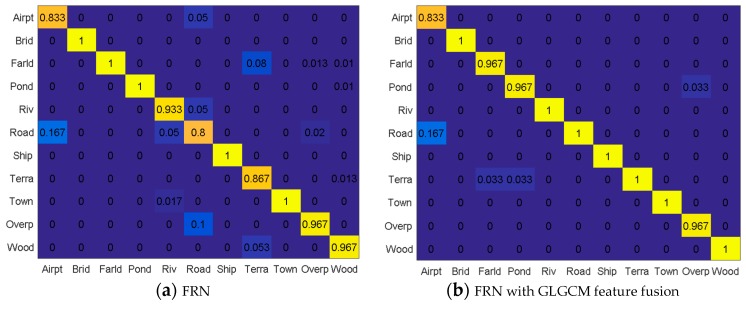

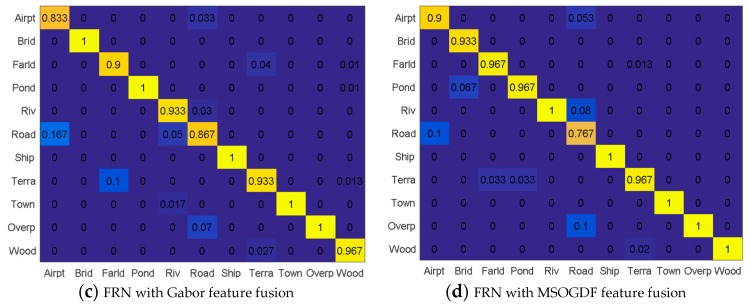
The confusion matrix of different methods for 11 types (‘Airpt’ means Airport, ‘Brid’ means Bridge, ‘Farld’ means Farmland, ‘Riv’ means River, ‘Terra’ means Terraced field, ‘Overp’ means Overpass, ‘Wood’ means Woods).

Four classification methods were tested based on the fused feature maps. They are FRN without feature extraction, FRN with the GLGCM feature, FRN with the Gabor feature, and FRN with the MSOGDF feature. The confusion matrices are shown in Figure 9. The abscissa axis shows the real 11 types of scene, while the ordinate shows the classified results of the 11 types. Compared with the standard FRN method, the remaining three methods presented better classification accuracy.

**Figure 10:**
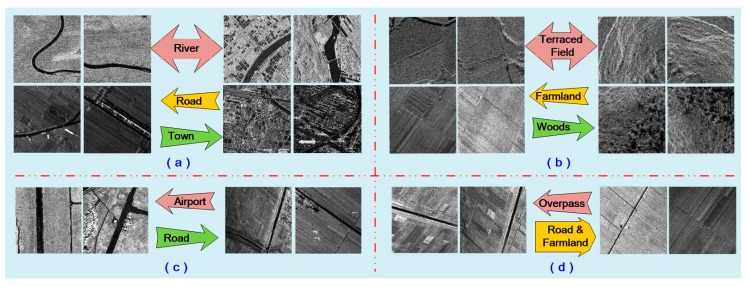
Some samples showing for terraced field, farmland and woods. (**a**) The looks alike scenes among Road, River and Town; (**b**) The looks alike scenes among Terraced Field, Farmland and Woods; (**c**) The look alike scenes between Airport and Road; (**d**) The look alike scenes between Overpass and Farmland.

From the FRN confusion matrix, we found that the classification accuracies of bridge, farmland, pond, ship and town were all 100%, because their features are relatively distinctive. While for the airport scene, 16.7% of the samples were classified as road due to their high similarity, which is illustrated in Figure 10c. For the river scene, there were 5% and 1.7% misclassifications as road and town, respectively. Possible reasons include the river might be embedded in a town, or the small river is very straight like a road, as samples depicted in Figure 10a. For the road scene, there were 10%, 5% and 5% misclassifications as overpass, airport and river. It might be because the road looks like a straight small river or overpass, or the road locates in the airport, such as the samples in Figure 10a,c,d. For the terraced field scene, there were 8% and 5.3% misclassifications as farmland and woods, because of their similar texture delineated in Figure 10b. For the overpass scene, there were 2% and 1.3% misclassifications as farmland and road; while the woods scene results in 1.3%, 1% and 1% misclassification as terraced field, farmland and pond, as shown in Figure 10b.

Compared with the FRN algorithm, the FRN with GLGCM feature fusion method improved the accuracies of river, road, terraced field and woods to 100% in our case study, which verifies the advantage of GLGCM considering the local gray level and gray gradient, which provides a better way to encode a scenes’ texture. However, we find the accuracies of farmland and pond were both reduced by 3.3%. They were misclassified as terraced field, and GLGCM feature maps of some farmland and pond samples are quite like features of terraced field samples. According to Figure 9c, the accuracies of road, terraced field and overpass increased by 6.7%, 6.6% and 3.3%, respectively, compared to FRN, but the accuracy of farmland decreased by 10%. After the MSOGDF feature fusion, the accuracies of airport, river, terraced field, overpass and woods were improved by 6.7%, 6.7%, 10%, 3.3% and 3.3% compared with FRN. However, the accuracies of bridge, farmland, pond and road decreased by 6.7%, 3.3%, 3.3% and 3.3%, respectively. 

From the above analysis, we conclude that the proposed framework using three methods of feature extraction and fusion are not suitable for every scene. Therefore, we should select an optimal method for different scenes according to the confusion matrix in Figure 9. The selected feature extraction methods for the 11 types of scene from Gabor, GLGCM, MSOGDF are presented in Table 4. In this table, 1 denotes the feature is selected for the scene classification, and 0 denotes the opposite. For the airport, the MSOGDF feature was selected, since it is the only feature that could improve the classification according to the experiment performance. For the bridge classification, Gabor and GLGCM features were selected with an identical weight, because the MSOGDF feature will increase the risk of misclassification into ponds. For the farmland, none of the features were selected, because they will all reduce the classification accuracy at least by 3.3%. For the pond, the Gabor feature was selected. For the river, the GLGCM feature and MSOGDF outperform. For the road, we prefer the GLGCM feature. Ships are very distinctive thus we do not need to employ feature extraction and fusion. For the terraced field, the GLGCM feature was the best. According to Figure 9, we found that the town’s classification accuracies were all 100% in the four confusion matrices, but there were some river samples which were misclassified as town samples, as show in Figure 9a,c. Therefore, GLGCM feature and MSOGDF features were selected for the town. For the overpass, Gabor and MSOGDF features were chosen. For the woods, GLGCM and MSOGDF features were adopted.

**Figure 11:**
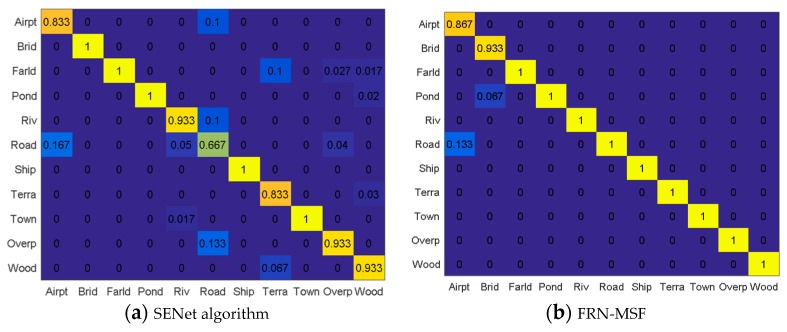
The confusion matrices for two algorithms. (**a**) SENet algorithm, (**b**) FRN-MSF.

Using the features fusion method in Table 4 and the proposed framework FRN, the final classified accuracies for the 11 types are shown in Figure 11b. Figure 11a lists the classified accuracy by using the SENet algorithm [36] without feature fusion, which achieved a better scene classification result than many new deep learning networks, such as ResNet-200 [53], Inception-V4 [54], ResNetXt-101 [55], DenseNet-264 [56] and PyramidNet-200 [57].

Compared with the SENet algorithm, the classification accuracy of the proposed framework outperformed. Our framework achieved 100% accuracy in 9 types of scene, and the accuracy of airport was improved by 3.4% as well. Only the accuracy of bridge was reduced by 6.7%. It is likely our fused features make some bridges samples more like pond samples. According to the classification results of FRN-MSF, some airport samples were classified as road. Table 5 gives the classification Mean Accuracy (MA) of different algorithms. FRN-Gabor denotes the FRN with Gabor feature, FRN-GLGCM means the FRN with GLGCM features, FRN-MSOGDF stands for FRN with MSOGDF features, and FRN-MSF is the proposed framework, which is FRN with multi-scale spatial statistical features. From Table 5 we can see that the proposed framework has achieved the best accuracy, which was improved by 6.07% in the whole compared with the SENet algorithm.

#### 4.3.3. Impacts of the Training Sample Ratios on the Performance

To find out the best training samples ratio, we repeated the experiments using our prior knowledge and physical accessibility and got the final accuracies for the 11 types of scene when the training sample ratios were 40%, 50%, 60%, 70%, 80% and 90%. We tested two algorithms for the experiments with the same datasets, FRN algorithm and FRN-MSF. Table 6 gives the mean accuracies of the two algorithms at different ratios.

**Figure 12:**
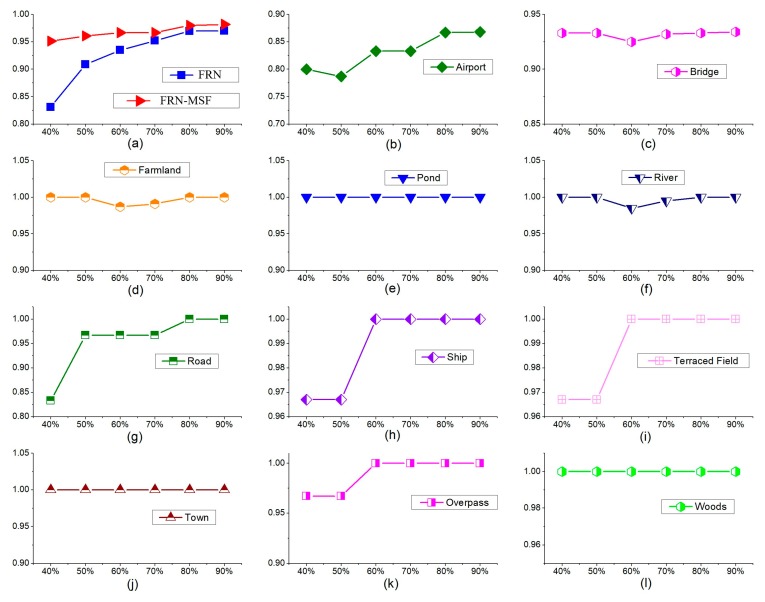
The classification mean accuracy with different training sample ratios. (**a**) The MA curves for FRN and FRN-MSF with training sample ratios; (**b**) The MA curves for Airport with training sample ratios; (**c**) The MA curves for Bridge with training sample ratios; (**d**) The MA curves for Farmland with training sample ratios; (**e**) The MA curves for Pond with training sample ratios; (**f**) The MA curves for River with training sample ratios; (**g**) The MA curves for Road with training sample ratios; (**h**) The MA curves for Ship with training sample ratios; (**i**) The MA curves for Terraced Field with training sample ratios; (**j**) The MA curves for Town with training sample ratios; (**k**) The MA curves for Overpass with training sample ratios; (**l**) The MA curves for Woods with training sample ratios.

From Table 6, we find the classification accuracy was basically no longer higher when the ratio exceeded 80%. Figure 12 shows the specific classification accuracy for the 11 types of scene with different training sample ratios. The abscissa axis stands for training samples ratios, from 40% to 90%; the ordinate shows corresponding classification accuracies. Figure 12a shows the mean classification accuracy for FRN and FRN-MSF with various training samplings ratios, with details being listed in Table 6. Figure 12b-(l) shows mean classification accuracies for the 11 types of scene. According to Figure 12, once the training samples ratio reached over 80%, there was only very limited accuracy improvement. Therefore, 80% of the total samples for each type were selected as the training samples in this paper, and with the rest of samples being used for testing.

#### 4.3.4. Generalization

**Figure 13:**
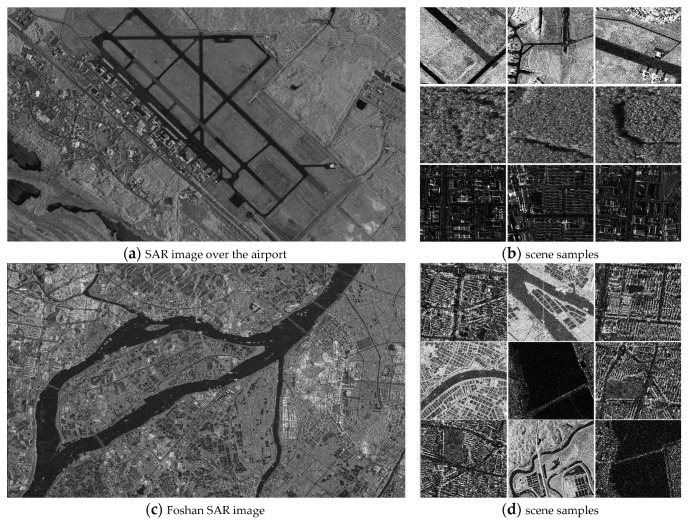
The SAR images and the samples acquired from the left SAR images.

To further validate the generalization ability of the proposed framework, we selected the two unused SAR images, Foshan SAR image from TerraSAR-X and an airport image (Carstensen airport) from Gaofen-3, which are shown in Figure 13. We designated five types of scene in total, which were river, town, bridge, airport and woods. Some of the samples are shown in Figure 13b,d. For each type, we collected 200 samples. Then, we use the model trained by the proposed framework to classify the 1000 samples for the five types.

**Figure 14:**
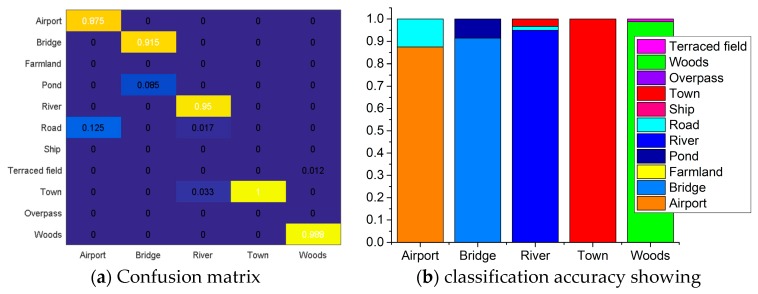
The classification results for the five types of scene.

Figure 14 delineates classification results for the five types of scene. From the figure, we found that the classification accuracy for the airport was 87.5%, and the remaining 12.5% was classified as road. For the bridge, the achieved accuracy is 91.5%, with 8.5% pond misclassification. The classification accuracy of rivers reached 95%, and the remaining 3.3% and 1.7% were classified as town and road, respectively. Fortunately, samples of town were all classified correctly, and 98.8% of the wood’s samples were classified correctly. The mean classification accuracy of the five types of scene was 94.56%, which was a little lower than the tested accuracy of 98.18% with the 11 types of scene in Table 6. When we recalculated the tested accuracy for the same five types of scene using the classification results in Figure 11b, we got a mean accuracy of 96%. Because the airport and bridge scenes are more challenging than the other types. Therefore, the mean classification accuracy of the validation data was 1.44% lower than the tested data, which is negligible considering the size of data. Consequently, the proposed framework FRN-MSF presented an excellent generalization ability for SAR scenes classification.

## 5. Discussion

In view of the three problems in SAR scene classification mentioned in the Introduction, we studied the second and third problems. We constructed a framework (FRN-MSF) for SAR scene classification which was not limited by resolutions and bands. It integrates GLGCM, Gabor transformation and MSOGDF to extract multi-scale space statistical features. To fuse these features, FRN-GLGCM, FRN-Gabor and FRN-MSOGDF are performed, from which different weights are selected for the three features according to the classification accuracy of different scenes. However, the selected weights might not be optimal. Therefore, it will be part of our future research on the method for choosing optimal weights automatically to further improve the algorithm. Furthermore, we found that over 10% of the airport samples were classified as road, due to their high similarity. Thus, we need to incur additional contextual information to enhance the performance of our FRN-MSF framework, which will be another focus of our future work. In addition, the first problem mentioned in the introduction (the module for speckle noise suppression) is also part of our following research, which will be very interesting and meaningful.

## 6. Conclusions

This paper proposed a new FRN-MSF framework for SAR scene classification. It first relies on multi-scale space statistical features (MSF) for feature extraction and fusion, then employs the proposed Feature Recalibration Network (FRN) to extract the high-level features, which are handled by FC neural networks for scene classification. In this paper, the proposed FRN integrates the Depthwise separable convolution, attention mechanism and CNN to improve the training speed and classification accuracy.

As far as we know, there are few SAR scene datasets provided in public except MSTAR datasets which are used for targets recognition. To test our proposed framework, we used scene samples for 11 types from various large-scale SAR images with different resolutions and bands. We used nine large-scale SAR images in total, including two TerraSAR-X images, five Gaofen-3 images, one MM-InSAR image and one CAS-InSAR image. Among them, seven SAR images were used to make samples for the 11 types for training and testing the framework. The remaining two large-scale SAR images (one TerraSAR-X image and one Gaofen-3 image) were used to evaluate the generalization ability of the framework. According to the experiment results, the classification mean accuracy reached 98.18% for the 11 types, which is 6.07% higher than the SENet algorithm. In the experiment of generalization evaluation, the mean classification accuracy could reach 94.56%, which was only 1.44% lower than the mean accuracy for the same five types in the former training and testing experiment. The proposed framework shows high accuracy and good generalization ability for SAR scene classification. It can be widely used for SAR scene classification without much consideration of the resolutions or the bands of the SAR systems. Moreover, it has shown a wide range of potential in target detection for large-scale SAR images.

## Figures and Tables

**Table 1:** SAR image datasets used in this study.

System	Platform	Band	Resolution (m)	Size(pixels)	Location (China)
TerraSAR-X	Satellite	X	3	14,804 × 30,623	Dongtinghu, Foshan
Gaofen-3	Satellite	C	1	31,699 × 26,193	Airports (i.e., Shanghai)
MM-InSAR	Airborne	Millimeter	0.15	10,240 × 13,050	Xi’an
CAS-InSAR	Airborne	X	0.5	16,384 × 16,384	Weinan

**Table 2:** Parameters setting of the network FRN.

Layer Types	Convolutions/Pooling Window Size
Input	−
Conv2D Convolution Layer C1	5 × 5 × 96
Pooling S1	2 × 2
SE Block_1	−
Conv2D Convolution Layer C2	3 × 3 × 256
Pooling S2	2 × 2
SE Block_2	−
Conv2D Convolution Layer C3	3 × 3 × 256
Pooling S3	2 × 2
NSE Block_1	−
Conv2D Convolution Layer C4	3 × 3 × 512
Pooling S4	2 × 2
NSE Block_2	−
FC_1	1024
FC_2	1024
FC_3	11
Softmax	−

**Table 3:** Average classification accuracy in three angles for MSOGDF.

Group	45° Group	90° Group	135° Group
Average Accuracy	95.45%	94.24%	94.55%

**Table 4:** Feature selection.

	Gabor Transformation	GLGCM Feature	MSOGDF Filter
Airport	0	0	1
Bridge	1	1	0
Farmland	0	0	0
Pond	1	0	0
River	0	1	1
Road	0	1	0
Ship	0	0	0
Terraced field	0	1	0
Town	0	1	1
Overpass	1	0	1
Woods	0	1	1

1: denotes the feature is selected; 0: denotes the feature is not selected.

**Table 5:** Classification accuracy of different algorithms.

Algorothm	MA (%)
SENet	92.11
FRN	94.24
FRN-Gabor	94.85
FRN-GLGCM	97.58
FRN-MSOGDF	95.46
FRN-MSF	98.18

**Table 6:** Different training samples ratios and classification accuracies.

Training Sample Ratio	40%	50%	60%	70%	80%	90%
Classification MA of FRN	89.09	91.81	92.72	93.33	93.94	93.95
Classification MA of FRN-MSF	95.15	96.07	96.67	96.67	98.18	98.20
